# Regulation of Lipid Metabolism by Lamin in Mutation-Related Diseases

**DOI:** 10.3389/fphar.2022.820857

**Published:** 2022-02-25

**Authors:** Yue Peng, Qianyu Tang, Fan Xiao, Nian Fu

**Affiliations:** ^1^ The Affiliated Nanhua Hospital, Department of Gastroenterology, Hunan Provincial Clinical Research Center of Metabolic Associated Fatty Liver Disease, Hengyang, China; ^2^ The Affiliated Nanhua Hospital, Clinical Research Institute, Hengyang Medical School, University of South China, Hengyang, China

**Keywords:** nuclear lamins, lipid metabolism, human diseases, mutation of lamin, lipodystrophy

## Abstract

Nuclear lamins, known as type 5 intermediate fibers, are composed of lamin A, lamin C, lamin B1, and lamin B2, which are encoded by *LMNA* and *LMNB* genes, respectively. Importantly, mutations in nuclear lamins not only participate in lipid disorders but also in the human diseases, such as lipodystrophy, metabolic-associated fatty liver disease, and dilated cardiomyopathy. Among those diseases, the mechanism of lamin has been widely discussed. Thereby, this review mainly focuses on the regulatory mechanism of the mutations in the lamin gene in lipid alterations and the human diseases. Considering the protean actions, targeting nuclear lamins may be a potent therapeutic avenue for lipid metabolic disorders and human diseases in the future.

## Introduction

**FIGURE 1:**
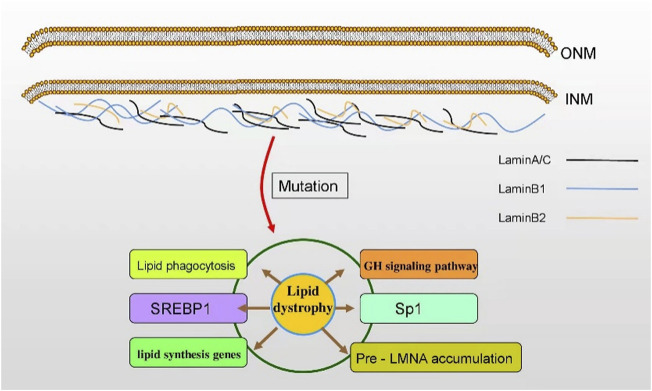
The subtypes and roles of lamin. Lamin A/C, lamin B1, and lamin B2 are the fibrin network structures located at the lower layer of the inner nuclear membrane. Lamin is responsible for maintaining the nuclear shape, participating in signal transduction, organizing chromatin, repairing DNA, and inducing apoptosis. The mutation of LMN genes affect lipid dystrophy in various human diseases. INM: inner nuclear membrane; ONM: outer nuclear membrane.

**FIGURE 2:**
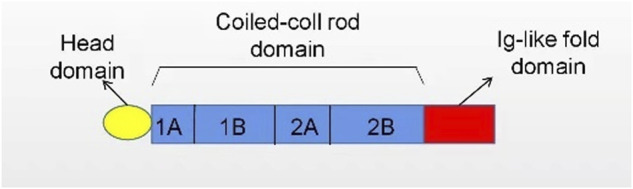
The structure of lamin. Nuclear lamins contain three domains: a head domain, a coiled-coil rod domain, and an Ig-like fold domain.

The nuclear lamina (NL) is fibrin network structure located in the lower layer of nuclear membrane, which is primarily composed of lamin A, C, B1, and B2 in mammalian cells. Lamins A and C are classified as type A encoded by *LMNA* while B1 and B2 as type B encoded by *LMNB1* and *LMNB2*, separately (Figure 1). As a major component of the nuclear lamina, lamin is responsible for maintaining the nuclear shape, transducting signals, organizing chromatin, repairing DNA, and pyroptosis (de Leeuw et al., 2018; Stiekema et al., 2020). In structure, lamin, which serves as a V-type intermediate filaggrin (IFS), forms the main cytoskeleton of the nucleus. Like all IFS, a V-type IFS has three components, mainly including an amino acid domain at the head, a helix domain at the center, and a carboxy-terminal domain at the tail. Additionally, the unique characteristics of these subcomponents mainly include a nuclear localization signal (NLS), IG folding domain, and CaaX motif (C = cysteine, A = aliphatic residue, X = any residue) (Gruenbaum and Medalia, 2015; Dittmer and Misteli, 2011) (Figure 2). Surpringly, the CaaX motif is a vital post-translational modification site for lipid metabolism (Kuchay et al., 2019). Therefore, considering the above structure of lamin, lamin may be an indispensable part in maintaining lipid homeostasis.

Indeed, the structure of lamin is of great importance to lipid metabolism. The dysfunction of lamin leads to numerous pathologies, mainly affecting the structure of the nuclear membrane, lipid synthesis genes, transcription factors, degenerative pathology, fat distribution, malnutrition, and aging (Östlund et al., 2020; Kim et al., 2018; Afonso et al., 2016; Ruiz de Eguino et al., 2012; Padiath and Fu, 2010). So, the abnormity of lamin elicits a series of metabolic disorders, the most common of which is lipodystrophy (Maraldi et al., 2011). The correlation between lamin and lipid metabolism has been well elucidated. For instance, the overexpression (OE) of lamin B1 downregulates the expression of lipid synthesis genes and the content of myelin-enriched lipids, ultimately increasing the risk of autosomal dominant leukodystrophy (ADLD) (Östlund et al., 2020). Also, NF-κB is activated by lamin A/C, subsequently boosting proinflammatory genes, such as Il6, Tnf, Ccl2, and Nos2, and finally promoting the development of obesity-induced insulin resistance in adipose tissue macrophages (ATMs) (Kim et al., 2018). As lamin always interferes with lipid metabolism, it is plausible that lamin-mediated lipid disorders may be intimately correlated with human diseases. Indeed, accumulating evidence has demonstrated that the mutations in the lamin gene play an important role on human diseases. Lamin genes are susceptible to mutate, hundreds of which are correlated to the human diseases. Furthermore, it is noteworthy that 17% of those diseases are lipodystrophy. Lipodystrophy syndrome is a rare heterogeneous disease characterized by systemic or partial fat atrophy with metabolic complications, which include insulin resistance, diabetes mellitus (DM), female hyperandrogenia, fatty liver, and dyslipidemia. Then, numerous studies have shown that lamin mutations are of great importance in human lipodystrophy syndrome (Table 1). More specifically speaking, lamin A/C mutation, prelamin A maturation, and lamin B mutation or deregulation have been proven to be the reasons or significant related factors of human lipodystrophy syndrome (Guénantin et al., 2014; Laver et al., 2018). Since lamin is associated with various lipid-related physiological alterations, more emphasis should be placed on the mechanism of the nuclear lamins in human diseases. Herein, this review summarizes the molecular mechanisms of lamin mutation-associated diseases concerning lipid metabolism.

**TABLE 1:** Nuclear lamina-related diseases about genetic lipodystrophy syndromes.

Lipodystrophy type	Genetic mutation	Clinical phenotype
Familial partial lipodystrophy type 2 (FPLD2)	LMNA (151660 AD)	Gradual loss of fat from the limbs and trunk, “cushingoid” appearance due to neck and face sparing, muscular dystrophy, dilated cardiomyopathy
Hutchinson–Gilford progeria syndrome (HGPS)	LMNA (176670 AD)	Generalized loss of subcutaneous fat, progeroid features
Mandibuloacral dysplasia with lipodystrophy (MAD type A)	LMNA (248370 AR)	Mandibular and clavicular hypoplasia, acro-osteolysis. Distal and truncal lipoatrophy, progeroid features
Mandibuloacral dysplasia with lipodystrophy (MAD type B)	ZMPSTE24 (608612 AR)	Mandibular and clavicular hypoplasia, acro-osteolysis. More generalized loss of fat, premature renal failure, progeroid features
Atypical Werner syndrome (AWS)	LMNA (150330 AR)	Partial or generalized loss of subcutaneous fat, progeroid features
Adult-onset demyelinating leukodystrophy (ADLD)	LMNB1 (169500 AD)	Downregulates the expression of genes associated with lipid synthesis, which in turn leads to a decrease in myelin-rich lipids
Acquired partial lipodystrophy (APL)	LMNB2 (608709 AD)	Gradual symmetrical subcutaneous fat loss, starting in the face and progressing down the upper part of the body. Subcutaneous fat in the lower abdomen and legs is significantly reduced, while fat storage in the gluteal area and lower limbs tends to be retained or increased

## The Regulatory Mechanisms in Human Diseases by Mutation of Lamin Genes

In the human disease spectrum, hundreds of mutations in the *LMNA* gene have been identified and are associated with more than a dozen human diseases, especially including lipodystrophy (Gonzalo et al., 2017). Lipodystrophy is a series of heterogeneous diseases characterized by the loss of selective adipose tissue or loss of functional adipose cells, thus leading to dyslipidemia and heterotopic steatosis (Hafidi et al., 2019). Specifically speaking, autosomal dominant mutations in *LMNA* genes are strongly correlated with familial partial lipodystrophy type 2 (FPLD type 2) (Hegele et al., 2000; Jéru et al., 2017). Additionally, the autosomal recessive mutation of *LMNA* gene is related to mandibuloacral dysplasia (MAD), which causes changes in lipid metabolism (Bagias et al., 2020). Besides, the mutation of LMN gene also elicits a dozen of diseases, including FPLD, Hutchinson–Gilford progeria syndrome (HGPS), metabolic associated fatty liver disease (MAFLD), MAD, dilated cardiomyopathy (DCM), autosomal dominant leukodystrophy (ADLD), acquired partial lipodystrophy (APL), Barraquer–Simons (BSS) syndrome, atypical Werner syndrome (WS), limb girdle muscular dystrophy type 1b (LGMD1B), and the autosomal dominant form of Emery–Dreifuss muscular dystrophy (AD-EDMD). In recent years, the regulatory mechanism of lamin mutation has been gradually elucidated. However, the understanding of the regulatory mechanism of mutated lamin in human diseases is not entirely clear, which is still being explored. The study on the regulatory mechanism of lamin mutation is helpful to reveal the importance of lamin in human diseases.

## LaminA/C Mutation-Related Diseases

### The Mutation of *LMNA* Increases Prelamin A Accumulation

FPLD2, a large genetic and phenotypic variation first reported in the 1970s, is characterized by the progressive loss of subcutaneous adipose tissue in the limbs and trunk, accumulation of fat in the face and neck, and severe metabolic disorders, including insulin resistance, glucose intolerance, diabetes, dyslipidemia, and steatohepatitis (Dunnigan et al., 1974; Köbberling et al., 1975; Krawiec et al., 2016). Interestingly, *LMNA* R482W and R482Q are common pathogenic variants in FPLD type 2 (Özen et al., 2020). FPLD and HGPS both belong to premature aging diseases and exhibit a significant loss of subcutaneous adipose tissue. More intriguingly, the pathogenesis of HGPS is caused by the *LMNA* mutation, preventing the conversion of prelamin A to mature lamin A, thereby leading to the accumulation of prelamin A. Later, a large number of studies reported the pathogenesis of lipodystrophy in FPLD roots in the accumulation of prelamin A by mutated *LMNA*, which is similar to the pathogenesis of HGPS (Capanni et al., 2005; Bidault et al., 2013; Afonso et al., 2016).

Conversely, Tu et al. (2016) doubted that most of the mutated sites of *LMNA* mutation in FPLD are not located in the key sequence for lamin A processing. In addition to this, some of these commercial antibodies bind nonspecifically to other proteins. To reconfirm the mechanisms involved, the monoclonal antibodies against prelamin A made by Tu were used for four subjects with *LMNA* mutations in lipodystrophy. Surprisingly, no evidence of prelamin A accumulation was found. As a result, Tu et al. suggested that the missense mutations of *LMNA* in FPLD cannot lead to an accumulation of prelamin A.

Meanwhile, a growing number of studies have shown that the function of lamin not only at the cellular level but also in disease states is controlled by the PTMs of proteins, including phosphorylation (Machowska et al., 2015), SUMOylation (Moriuchi et al., 2016), glycosylation (Snider and Omary, 2014), farnesylation (Farnsworth et al., 1989), methylation (Rao et al., 2019), o-glcNAcylation (Alfaro et al., 2012; Wang et al., 2012; Simon et al., 2018), succinylation (Weinert et al., 2013), and ubiquitination (Wagner et al., 2011; Povlsen et al., 2012). Farnesylation is special among many PTMs in the regulation of prelamin A. Specifically speaking, pre-*LMNA* (the precursor of mature *LMNA*) and B-type LMN are a fannification on the cysteine residues of carboxy-terminal-Caax motifs (Weber et al., 1989). Moreover, the three terminal amino acids on type A and type B LMN were subjected to zinc metalloproteinases (ZMPSTE24; prelamin A) or RAS-converting enzyme 1 (Rce1; *LMNB1* and B2) and cleaved by methylated *α* -carboxyl groups (Winter-Vann and Casey, 2005). Interestingly, *LMNC* is not able to be fannized due to lack of the -Caax motif (Goldberg et al., 2008; Adam et al., 2013; Jung et al., 2013). Therefore, ZMPSTE24 is essential for prelamin A to become a mature lamin A. Afonso et al. (2016) discovered that the expression of ZMPSTE24 decreases in FPLD cells, but it is not clear why *LMNA/C* missense mutation affected the expression of ZMPSTE24. Therefore, for lipid dystrophy caused by *LMNA* mutation in FPLD, does *LMNA* mutation cause prelamin A accumulation? If there is an accumulation of prelamin A, is it caused by *LMNA* mutation that reduces the expression of ZMPSTE24?

In short, there is much evidence that the accumulation of prelamin A by mutated *LMNA* causes FPLD and HGPS. Since prelamin A has a paradoxical role in the occurrence and development of FPLD and HGPS, the relevant regulatory mechanism of *LMNA* mutation remains to be further explored.

### The Mutated Lamin Elicits Dynamic Recombination of Nuclear Layer Networks

The rupture of lamina is an early event and a prerequisite of lipogenesis and lipocyte differentiation. To understand the underlying regulatory networks, Verstraeten et al. (2011) have found broken lamina, the loss of lamin, and emerin proteins at the 10th day of fat cell differentiation. Eight days after that, the proportion of cells expressing lamins increase while lamin A/C protein levels remain low in the whole. Thus, the re-expression of lamin subtypes increases the plasticity of nuclear membrane to indentations under lipid stress, ultimately causing a reorganization of the cellular infrastructure. Moreover, progerin, a farnylated protein resulting from *LMNA* mutation, can harden the nucleus and reduce the reorganization, finally wiping the differentiation of lipocytes. Lipid accumulation happens, while low progerin expresses (Najdi et al., 2021). In short, targeting progerin may be an effective method to improve lipid disorders.

### The Mutation of *LMNA* Enhances Lipophagy

Lipophagy is mainly manifested as the interaction of the autophagosome membrane with LC3. Then, the lipid droplets (LDs) are selectively delivered to the lysis chamber for degradation by the autophagic protein (Singh and Cuervo, 2012; Wang, 2016; Kloska et al., 2020; Shin, 2020). In a recent study, Chad A. Cowan et al. (Friesen and Cowan, 2018) discovered that in lipolysis, the proportion of LC3-II and LC3-I, the level of ATG7 protein significantly stimulates LMNA R482W mutant cells, thereby indicating that autophagosome formation increases in FPLD2 adipocytes. As described above, reduced fat production, increased lipolysis, and increased autophagy may be intimately related to the lipid abnormalities of FPLD2. As a result, *LMNA* mutation promotes lipophagy, while the deeper connection between the lamin gene and lipophagy needs more exploration.

### The Lamin A/C Activates Liver Growth Hormone Receptor Signals

MAFLD, a clinicopathological syndrome, is characterized by an excessive deposition of fat in liver cells caused by non-alcohol and other clear liver damage factors and is closely related to metabolic stress liver injury (Mantovani and Dalbeni, 2020). Recently, Vargas et al. (Mahdi et al., 2020) indicated that a case of MAFLD patient was derived from the D300N *LMNA* mutation of FPLD, which surprisingly suggested that the mutation of the lamin gene may progress to steatosis, therefore eliciting MAFLD. Accordingly, the subsequent genetic testing and the risk of MAFLD should be taken seriously in FPLD-diagnosed patients. Beyond that, the change of lamin protein may also contribute to the occurrence and development of MAFLD. Recent research reported that the specific lamin A/C deficiency of hepatocellular in mice induces spontaneous liver injury and increases the susceptibility to steatohepatitis fed with high-fat diets in mice (Kwan et al., 2017). Considering the fact mentioned above, it is plausible that lamin protein is intimately correlated with MAFLD.

**FIGURE 3:**
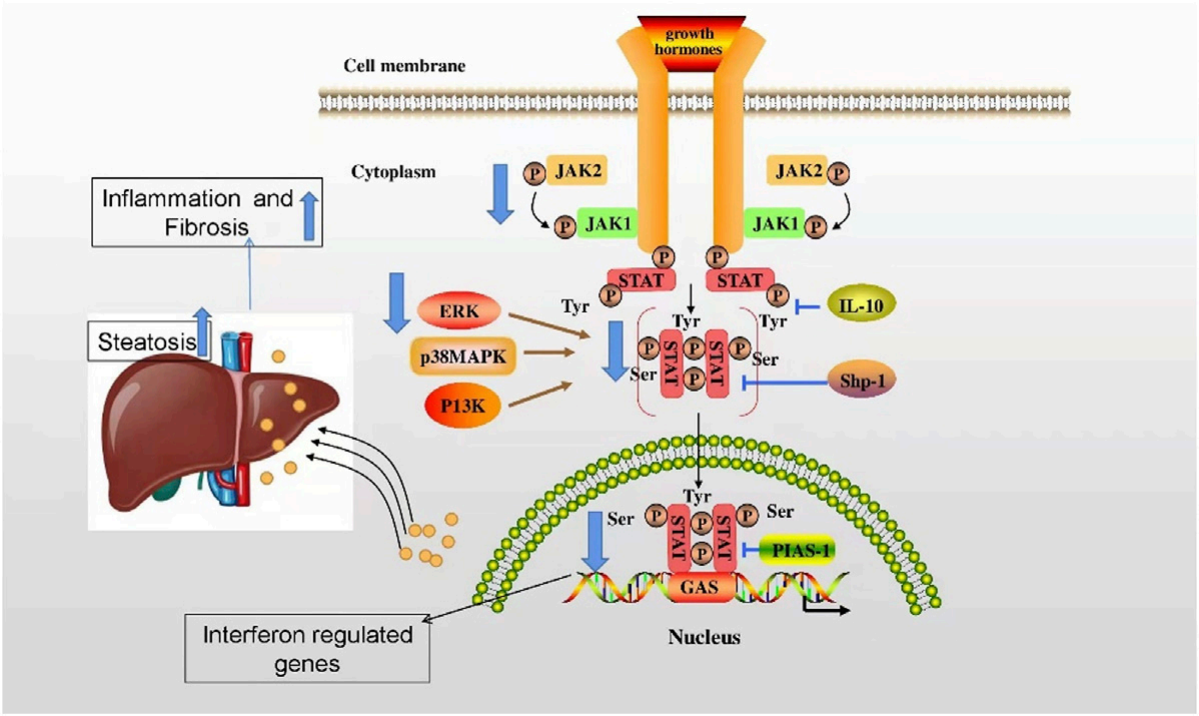
The regulatory mechanism of lamin in alleviation of MAFLD. The deficiency of lamin A/C upregulates stat1 mRNA and protein levels and blocks JAK2, Stat 5, and ERK mediated by the liver GH receptor signal, thus downregulating the expression of stat5-dependent male-specific genes, ultimately promoting excessive fatty acids, inflammation, and fibrosis in hepatocytes and exacerbating the progression of MAFLD.

Indeed, the regulatory mechanism of lamin in MAFLD has been gradually elucidated. Nevertheless, the understanding of the regulatory mechanism remains unclear. It has been found that the deficiency of lamin A/C upregulates stat1 mRNA and protein levels and blocks the phosphorylation of Janus kinase 2 (JAK2), transcription activator (Stat 5) and extracellular regulated protein kinases (ERKs) mediated by the liver growth hormone (GH) receptor signal, thus downregulating the expression of stat5-dependent male-specific genes, ultimately promoting excessive fatty acids, inflammation, and fibrosis in hepatocytes and exacerbating the progression of MAFLD (Kwan et al., 2017) (Figure 3).

In general, the mutation of the *LMNA* gene induces MAFLD. Similarly, the GH signal pathway and stat1 mediated by lamin A/C play protective roles in delaying MAFLD progression. Nevertheless, the regulatory mechanism of lamin in MAFLD remains to be further studied.

### Prelamin A Segregates SREBP1 at Nuclear Margin

MAD, an extremely rare autosomal recessive disorder, is mainly manifested as bone abnormalities, premature aging, and lipodystrophy (Bagias et al., 2020). Growing evidence suggests that the lamin mutation may be a vital part in the lipid changes of MAD. Firstly, a study hinted that a single amino acid substitution in laminin A/C causes MAD (Novelli et al., 2002). In addition, the two defects in MAD are the mutation of *LMNA* or ZMPSTE24 genes and are named as type A or B MAD, respectively. Intriguingly, it has been found that the different types of MAD also cause various fat changes. Specifically speaking, type A MAD (MADA) loses fat in the extremities, while fat deposits in the neck and trunk are normal or excessive; type B MAD (MADB) forms subcutaneous fat loss that resulted from a typical mutation in the ZMPSTE24 gene (Cenni et al., 2018). The above evidence shows that the mutated lamin is intimately correlated with lipid disorders in MAD. Moreover, some studies have demonstrated that the regulatory mechanism of prelamin A plays an important role in MAD.

Indeed, some studies have demonstrated that prelamin A plays an important role in regulating lipid homeostasis. Lattanzi et al. (Capanni et al., 2005) reported that prelamin A accumulated in Dunnigan familial partial lipodystrophy, mandibuloacral dysplasia, and atypical Werner syndrome, which are three laminopathies characterized by lipodystrophic phenotypes. Furthermore, prelamin A precursors specifically accumulate in dystrophic cells and lipodystrophic cells and colocalize with cholesterol regulatory element binding protein 1 (SREBP1) (SREBP1: The key regulators of lipid metabolism, involved in adipocyte differentiation, are expressed at high levels in adipose tissue, and stimulates the expression of a variety of adipogenic genes, including FAS,acetyl-CoA carboxylase, stearoyl-coenzyme A desaturase1, and lipoprotein lipase (Li et al., 2016). Lattanzi et al. also suggested that not mature lamin A/C but rather prelamin A interacts with SREBP1. The mechanism is that prelamin A isolates SREBP1 at the nuclear border, thereby declining the activation of peroxisome proliferator activated receptor gamma (PPAR gamma) *via* inhibiting active SREBP1, thus impairing pre-adipocyte differentiation (Capanni et al., 2005).

In conclusion, the lamin mutation leads to lipid changes in MAD. Also, prelamin A interacts with SREBP1, thus affecting the actions of pre-adipocytes. However, the understanding of the lipid-regulated mechanism of lamin is not entirely clear, which is still being explored.

### Prelamin A and Sp1 Effects on Adipogenesis

To investigate the effect of lamin on transcriptional factors, research shows that lipodystrophy can not only result from lamin mutation but also from the use of human immunodeficiency virus protease inhibitors (PIs). Under PI treatment in human mesenchymal stem cells (hMSC), impaired adipogenesis is due to an interaction between accumulated prelamin A and Sp1 transcription factors, finally altering extracellular matrix gene expression (Ruiz de Eguino et al., 2012). In general, the interaction between prelamin A and Sp1 excacerbates *LMNA*-linked lipostrophy. Nevertheless, the regulatory mechanism between lamin and transcriptional factors in human diseases remains to be further studied.

### The Activation of Tumor Protein 53

DCM is characterized by a progressive conduction system disease, arrhythmia, and systolic impairment (Captur et al., 2018). It has been studied that cyclin-dependent kinase inhibitor 2A (CDKN2A), a downstream target of the E2F pathway, is responsible for activation. *LMNA* D300N, associated with DCM, results from E2F/DNA damage/TP53 activation. This axis can be a potential intervention target for DCM in laminopathies (Augusto et al., 2020). Furthermore, the impaired crosstalk between endothelial cells (ECs) and cardiomyocytes (CMs) can contribute to the pathogenesis of *LMNA*-related DCM (Sayed et al., 2020). The other sites of cardiac involvement in DCM contain missense lamin A/C mutation (Arg60Gly) (Porcu et al., 2021).

## LaminB1 Mutation-Related Diseases

### Lamin B1 Downregulates Lipid Synthesis Genes

ADLD is a slow-progressing but fatal neurological disorder in 40–50-year-old adults, usually accompanied by symptoms of autonomic nervous dysfunction, followed by ataxia and cognitive impairment and even the loss of myelin sheath in the central nervous system (CNS) (Chrast et al., 2011; Giorgio et al., 2015). Interestingly, ADLD is the only disease associated with the lamin B1 gene (Padiath, 2019). Otherwise, the pathogenesis of ADLD is overexpressed lamin B1 protein levels due to *LMNB1* gene replication or upstream deletion (Takamori et al., 2018). In addition, lamin B1 OE targets oligodendrocytes, thus decreasing the production of myelin sheaths in the CNS. Furthermore, Rolyan et al. also discovered that lamin B1 OE mouses exhibit severe demyelination, axon damage, and neuron loss due to the decreased gene expression of lipid synthesis pathways that play an important role in myelin regulation, ultimately depleting myelin-rich lipids (Rolyan et al., 2015). Indeed, the myelin genes required for oligodendrocyte maturation are sensitively influenced by the nuclear membrane (Lin et al., 2011). However, another study indicated that increased lamin B1 alters the chromatin associated with the region of the nuclear layer, therefore affecting the structure of the nuclear membrane and myelin-related genes (Padiath and Fu, 2010).

In conclusion, the expression of lamin B1 exacerbates ADLD. Despite considerable studies that are accessible, more experiments are necessitated about the lipid-related effects of lamin.

## LaminB2 Mutation-Related Diseases

### Overactivation of the Complement System

Mutations in the *LMNB2* have been associated with APL, also known as BSS syndrome, which usually begins in childhood or adolescence. Fat loss in BSS is typically characterized by gradual symmetrical subcutaneous fat loss, starting in the face and progressing down the upper part of the body. The subcutaneous fat in the lower abdomen and legs is significantly reduced, while fat storage in the gluteal area and lower limbs tends to be retained or increased (Hegele et al., 2006; Oliveira et al., 2016). CORVILLO et al. analyzed clinical, immunological, and histological events in an 11-year-old girl with BSS during a 5-year follow-up, and their results suggest that the overactivation of the complement system in adipose tissue may be responsible for fat loss in BSS patients (Corvillo et al., 2020).

## The Regulatory Mechanisms Are Still Unclear

WS or atypical WS is a rare autosomal recessive disorder caused by inherited mutations in the *WRN* gene and *LMNA* gene, respectively (Rossi et al., 2010; Wang et al., 2018). In atypical WS associated with the R133L mutation of the *LMNA* gene, the severity of metabolic complications is positively related to the degree of lipodystrophy (Doh et al., 2009). In addition, Garg et al. investigated the body fat distribution pattern and metabolic abnormalities in two patients with atypical WS carrying R133L heterozygous *LMNA* mutations. Both patients with *LMNA* mutations had a unique distribution of body fat, with patient 1 having a fat loss limited to the distal portion of the limbs and an increase in fat deposition in the trunk region, whereas patient 2 had a significant decrease in body fat. In summary, patients with atypical Werner syndrome caused by R133L heterozygous *LMNA* mutations may present with different types of lipodystrophies, which may present with partial or total body fat loss. Partial lipodystrophy can be further divided into two distinct patterns: one involves the entire limb, mainly including familial partial lipodystrophy, the Dunnigan type, and mandibular dysplasia, while the other happens to involve only the distal limb region (Muchir et al., 2000). However, the regulatory mechanism needs to be further discovered.

Emery–Dreifuss muscular dystrophy (EDMD) is a severe muscular disorder characterized by the early contracture of the elbows, slowly progressive muscle weakness, and cardiomyopathy with conduction block (Onishi et al., 2002). Lamin A/C defects occur both in X-EDMD and AD-EDMD (Niebroj-Dobosz et al., 2003). Lamin A is not only required for lamin B receptor (LBR) retention but also for the localization of transcriptional RNA pol II in muscle cells (Reichart et al., 2004).

Limb girdle muscular dystrophy (LGMD), a type of muscular dystrophy (MD), is manifested as the progressive weakness of muscles (Rajoria et al., 2021). Though the regulatory mechanism is still unclear, a survey has reported that the lipid changes of LGMD are similar to FPLD in skeletal muscle metabolism, mainly exhibiting incomplete fatty acid oxidation and upregulated ketogenesis, which may result from a common underlying cause of muscular metabolic disorders (Boschmann et al., 2010). Actually, LGMD1B is same as FPLD and EDMD (Morris, 2001). Besides, LGMD1B and AD-EDMD are allelic disorders (Muchir et al., 2000).

## Conclusion

As a component of nucleus, lamin plays an important role in maintaining nuclear shape, mechanical signaling, stabilizing chromatin, regulating gene expression, and promoting cell cycle progression. Here, we amply review the possible mechanisms of innate or acquired lipid abnormalities caused by lamin in the human diseases, mainly including increased prelamin A accumulation, a dynamic recombination of nuclear layer networks, enhanced lipophagy, activated liver growth hormone receptor signals, segregated SREBP1 at the nuclear margin, adipogenesis, lipid synthesis genes, overactivated complement system, and activated TP53. Those are the targets for lipid defects caused by lamin alterations. Nevertheless, the regulatory mechanism of lamin in lipid metabolism and human diseases remains to be further studied. As a result, targeting lamin should be considered for treating human diseases, which may be a promising disease-reversing strategy for patients.

## Prospection

Lipolysis and autophagy are two central catabolic pathways of lipid decomposition (Zechner et al., 2017). Lipolysis depends on the direct activation of lipase related to LDs, such as adipose triglyceride lipase (ATGL), hormone-sensitive lipase (HSL), and monoglyceryl lipase (MGL). In addition, LDs interact with ATGL activators and inhibitors and then provide energy and basic materials for the synthesis of cell membranes and hormones in the body (Onal et al., 2017). Another lysosomal autophagy pathway that plays an important role in lipid degradation is called lipid autophagy, or lipophagy for short. Lipophagy requires cargo identification accomplished by the interaction between the autophagosome membrane with LC3. Subsequently, LDs are selectively delivered to the lysis chamber for degradation by the autophagic protein (Singh and Cuervo, 2012; Wang, 2016; Kloska et al., 2020; Shin, 2020). Thus, the dysregulation of lipophagy can lead to an abnormal deposition of lipids, therefore seriously affecting cell function and dynamic balance, ultimately resulting in cell death and a variety of diseases, including non-alcoholic fatty liver disease, coronary heart disease, and even cancer (Johnson and Stolzing, 2019).

**FIGURE 4:**
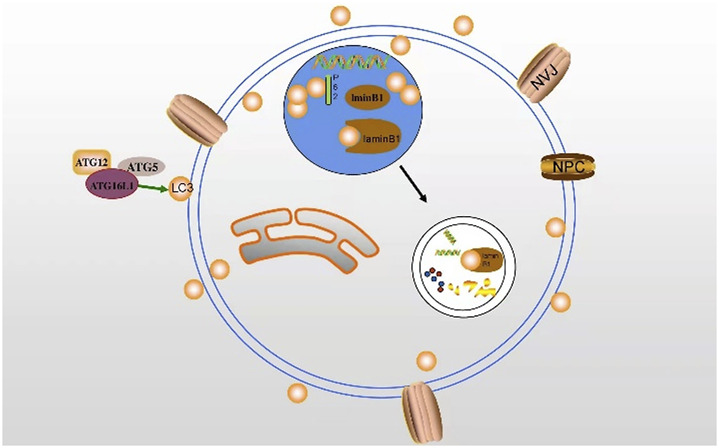
The interaction between LC3 and lamin B1 may induce nuclear autophagy. LC3 transports autophagy membrane and substrate *via* binding to lamin B1 in the nucleus.

Nuclear autophagy, a new type of selective autophagy, is responsible for selectively removing damaged or unnecessary nuclear substances in cells (Fu et al., 2018). Nuclear autophagy happens in a variety of conditions, including starvation, rapamycin-induced TORC1 inactivation, nuclear vacuolar junction (NVJ) expansion, and nuclear fibrillary lamina defects (Bo Otto and Thumm, 2020). In 2009, it was first reported that *LMNA/C* is involved in the development of mammalian nuclear autophagy. Additionally, some nuclear components exist in perinuclear autophagosomes and lysosomes (Park et al., 2009). Dou et al. also found a large amount of endogenous LC3 and a small amount of lipidated LC3-II in the nucleus. Lamin B1 interacts with LC3 to induce nuclear autophagy, which may be enhanced by lipidated LC3 (Dou et al., 2015) (Figure 4). Additionally, the NEM1-spo7/Pah1 axis is very important in the lipid synthesis axis. Meanwhile, this axis is also an important factor to induce nuclear autophagy and correct the localization of micronucleus autophagy factor NVJ1 and nuclear autophagy receptor Atg39 (Rahman et al., 2018; Mirheydari et al., 2020).

Combined with the above literature, it is not difficult to find that nuclear autophagy is significantly correlated with lipid metabolism. For instance, lamin participates in the occurrence and development of nuclear autophagy by interacting with LC3, and the latter one is an essential factor in the process of lipophagy. As a result, the nuclear autophagy and lipophagy involved in lamin may be not independent of each other, although the specific correlation mechanism and the relationship between lamin-dependent nuclear autophagy and lipid metabolism deserve to be further studied.

Apart from autophagy, apoptosis is another form of cell death. Apoptosis is characterized by caspase activation, DNA cleavage, and membrane surface modifications that enable apoptotic and phagocytic cells to be recognized, as well as morphological changes such as chromatin concentration and nuclear fragmentation (Jung et al., 2020a). Importantly, lamin also seems to act in the relationship between apoptosis and lipid metabolism. In nonadipose tissues such as liver, heart, kidney, muscle, and islet, the harmful effects caused by excessive accumulation of fat on these organs or systems are called lipotoxicity, which can induce programmed cell death, and lipid apoptosis is the main cellular consequence of lipotoxicity (Schaffer, 2016; Zhang et al., 2019). In all, the involvement of lamins in nuclear autophagy and apoptosis is also a promising point for lipodystrophy and deserves to be further explored in the future.

**FIGURE 5:**
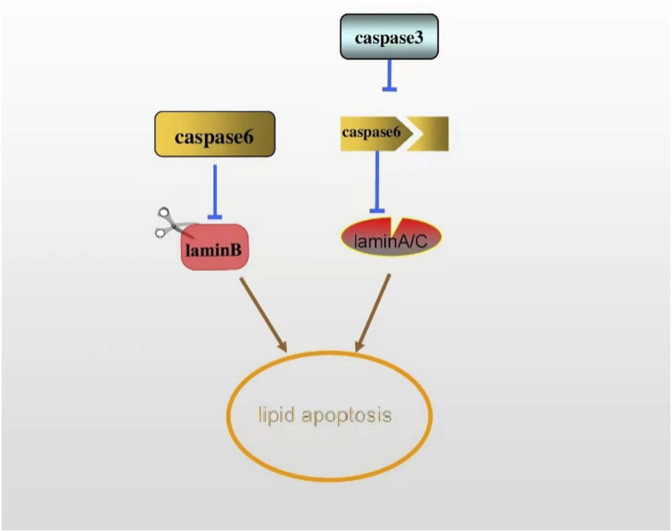
The early cleavage of lamin B by caspase-6 is regarded as a marker of apoptosis. Lamin A can also be a substrate of caspase-6. In apoptosis, caspase-6 is cleaved by caspase-3, thus cleaving lamin A/C, ultimately activating the apoptosis.

Chromatin concentration is a nuclear modification characteristic of the active apoptotic phase that follows DNA cleavage and the hydrolysis of certain nuclear proteins by proteases in the caspase family. The cysteine aspartate protease family (caspase) is the main factor affecting protein hydrolysis in the process of apoptosis. The apoptotic caspase can be divided into two types: the initiation of caspase, including caspase-8, -9, and -10, and the execution of caspase, including caspase-3, -6, and -7. Caspase-3 and caspase-6 are responsible for the cleavage of nuclear proteins PARP and lamin, respectively. Among many apoptosis-related proteins, the hydrolysis of PARP by caspase-3 is considered as an early indicator of apoptosis. The early cleavage and rapid processing of lamin B by caspase-6 are regarded as a marker of apoptosis (Villa et al., 1997; Buendia et al., 1999; Eron et al., 2017; Jung et al., 2020b). Meanwhile, lamin A can also be cleaved as a substrate of caspase-6. Studies have shown that caspase-3 cleaves caspase-6 first in normal cell apoptosis, and caspase-6 cleaves lamin A/C before apoptosis (Capo-Chichi et al., 2018). Only when lamin A/C is cleaved by caspase-6 can chromosomal DNA fully coagulate during apoptosis (Yan et al., 2021) (Figure 5). Lamin plays a key role in apoptosis, while the specific mechanism of the involvement of lamin in lipid apoptosis remains to be further studied. For instance, if adipocytes show apoptosis in lipodystrophy, then silencing caspase may prevent its interference with lamin expression, subsequently ameliorating lipid defects.

In MAD, we review the interaction between prelamin A and SREBP1, suggesting that prelamin A sequestrates SREBP1 at the nuclear border and restricts the translocation of some transcription factors into the nucleus, thereby reducing the pool of normally activated active SREBP1 and leading to the dysdifferentiation of adipocytes (Capanni et al., 2005). Meanwhile, it has been reported that SREBP1c (one of the subtypes of SREBP1) modified by SUMO1 (small ubiquitin-like modifier, also named SUMOylation, is a crucial post-translational modification that exhibits a strong effect on DNA repair, transcriptional regulation, protein stability, and cell cycle progression) can repress the transcriptional activity of SREBP1c and inhibits lipid production (Pichler et al., 2017; Zeng et al., 2020). Similarly, several lamin A domains can also be modified by SUMOylation. Two typical mutations cause lipid dystrophy (*LMNA* P.g465d and P.K486N), while only the atypical FPLD2-related p. r482W mutation shows a decrease in lamin A sumoylation. This may provide an alternative mechanism for these atypical lipodystrophies (Simon et al., 2013).

The distribution of adipose tissue is not entirely alike in various types of lipodystrophy. Moreover, the special distribution is an ongoing research. Additionally, the major foregone mutated genes in lipid disorders encode proteins forming lipid droplets (Meehan et al., 2016). However, not only the connections between genetic mutation and fat loss but also the cure strategies still need to be discovered. Metreleptin, a recombinant analog of human leptin, is the only drug approved for the treatment of metabolic complications associated with lipodystrophy. This compound is used for the replacement of lipodystrophy accompanied by leptin deficiency without HIV infection (Chevalier et al., 2021). Nonetheless, except for Japan, no country authorizes metreleptin as a drug for lipid issues. Worse, it is not entirely clear whether metreleptin is a benefit or not to lipodystrophy syndrome with normal leptin levels (Oral et al., 2019). Worse still, various complications in the treatment of lipodystrophy have shown to us all. Notably, the most common of which includes weight loss, abdominal pain, hypoglycemia, fatigue, headache, a loss of appetite, injection site reactions (bruising and hives), anti-leptin antibodies, T-cell lymphoma, and infection (Tchang et al., 2015). Even so, metreleptin treatment powerfully alleviates metabolic abnormalities such as hyperglycemia, hypertriglyceridemia, increased hepatic fat content, and elevated liver enzymes alanine transaminase and aspartate transaminase and corrects the hyperphagia of leptin deficiency in patients with generalized lipodystrophy (Akinci and Akinci, 2015).

Considering that there is no efficient and safe drug for curing lipodystrophy, targeting relevant mechanisms regulated by lamina may be a promising strategy. The perturbations in mutated lamin are closely associated with the lipid issues, and an understanding of the contribution and influence of lamins in human diseases poses an exciting area for scientific discovery.

## Glossary

ADLD: autosomal dominant leukodystrophy
AD-EDMD: autosomal dominant form of Emery–Dreifuss muscular dystrophy
APL: acquired partial lipodystrophy
ATGL: adipose triglyceride lipase
BSS: Barraquer–Simons syndrome
CDKN2A: cyclin-dependent kinase inhibitor 2A
CNS: central nervous system
CMs: cardiomyocytes
DM: diabetes mellitus
DCM: dilated cardiomyopathy
ECs: endothelial cells
EDMD: Emery–Dreifuss muscular dystrophy
ERK: extracellular regulated protein kinase
GH: growth hormone
HSL: hormone-sensitive lipase
hMSC: human mesenchymal stem cell
HGPS: Hutchinson–Gilford progeria syndrome
IFS: intermediate filaggrin
INM: inner nuclear membrane
JAK2: Janus kinase 2
LGMD: limb girdle muscular dystrophy
LGMD1B: limb girdle muscular dystrophy type 1b
LD: lipid droplet
LBR: lamin B receptor
MADA: type A mandibuloacral dysplasia
MADB: type B mandibuloacral dysplasia
MAD: mandibular sacral dysplasia
MAFLD: metabolic associated fatty liver disease
MD: muscular dystrophy
MGL: monoglyceryl lipase
NLS: nuclear localization signal
NVJ: nuclear vacuolar junction
ONM: outer nuclear membrane
OE: overexpression
PPAR gamma: peroxisome proliferator activated receptor gamma
PI: protease inhibitor
PTMs: post-translational modification
SUMOylation: small ubiquitin-like modifier
Stat5: transcription activator
SREBP1: cholesterol regulatory element binding protein 1
WS: Werner syndrome
ZMPSTE24: zinc metalloproteinase
